# Occult Hepatitis B Virus Infection Among Hemodialysis Patients

**DOI:** 10.5812/hepatmon.869

**Published:** 2012-04-30

**Authors:** Seyed Moayed Alavian

**Affiliations:** 1 Baqiyatallah Research Center for Gastroenterology and Liver Diseases, Baqiyatallah University of Medical Sciences, Tehran, IR Iran

**Keywords:** Hemodialysis, Viruses, Hepatitis B

Occult hepatitis B (OHB) is not a new entity and there are many reports of patients with hepatitis B virus (HBV) evidence replication in the absence of detectable hepatitis B surface antigen (HBsAg) and occasionally other HBV serologic markers [1]. After introducing highly sensitive and specific tests for HBs Ag and HBV DNA, the diagnosis of OBH is easier and the importance of clinical entity of OBH is more controversial. In this issue of Hepatitis Monthly, Abu El Makarem reported the prevalence of occult hepatitis B virus infection in hemodialysis patients from Egypt with considering the hepatitis C Virus (HCV) Infection [2]. Prevalence of HBV DNA detection in hemodialysis patients was reported 4.1%, and the authors concluded occult HBV infection (OBI) in those patients. They did not find any correlation between the variables and OBI. I would like to mention that finding the HBV DNA in the serum is not parallel to OBI and it is better to use the term of OHB instead of OBI in their cases. Moreover, in a literature review it has been suggested that occult HBV in patients with chronic HCV infection has a possible correlation with higher grades of liver damage or even developing hepatocellular carcinoma [3]. In addition, chronic HCV infection is frequently reported with occult HBV infection, due to almost the same route of transmission and risk factors [3].

Egypt has the highest prevalence of HCV infection in the world [4]. However the detection of OBH requires assays with the highest sensitivity and specificity and a lower limitation to detect less than 10 IU/ml HBV DNA [5]. It is very interesting for us that the authors declared they used the real time PCR kit (COBAS Taqman HBV Test; cutoff detection: 6 IU/mL) that is a very sensitive test in their study. As they presented, the prevalence of anti-HBc Ab as well as the history of HBV infection in group one (HCV RNA positive ones) was more common than group two (HCV RNA negative). This condition is relatively more common in patients with HCV infection that seems to be related to influence on the replicative capacity and latency of HBV and the main risk factors for airing these infections. The authors did not find any relation between the ALT level and the presence of HBV DNA in the serum, In my opinion the liver biopsy provides more relevant data than ALT in liver damage specifically in hemodialysis patients [6].

End-stage renal disease (ESRD) is a significant problem in almost all countries and the prevalence has increased considerably in developing countries especially in Middle East countries. The prevalence of HCV infection among hemodialysis patients differs in different parts of Middle East countries which is 48% in Egypt [7]. The prevalence of OBH in hemodialysis patients varies from 0% to more than 20% in different studies and it requires more investigations with more sample size to find out OBH impact in transmission of HBV infection in hemodialysis centers.
